# Occurrence, Ecological and Health Risk Assessment of Phthalate Esters in Surface Water of U-Tapao Canal, Southern, Thailand

**DOI:** 10.3390/toxics8030058

**Published:** 2020-08-17

**Authors:** Okpara Kingsley, Banchong Witthayawirasak

**Affiliations:** 1 Faculty of Environmental Management, Prince of Songkla University, Hat Yai, Songkhla 90112, Thailand; okingus07@yahoo.com; 2 Research Program of Municipal Solid Waste and Hazardous Waste Management, Center of Excellence on Hazardous Substance Management (HSM), Bangkok 10330, Thailand

**Keywords:** environmental contamination, noncarcinogenic and carcinogenic risk, risk quotient (RQ), endocrine disruption, outliers

## Abstract

Phthalate esters (PAEs) are well known for their environmental contamination and endocrine-disrupting effects on wildlife and humans. In this study, the occurrence of PAEs and ecotoxicological risk assessments were performed in one of the significant canals in southern Thailand, named U-Tapao. Water samples were collected and analyzed for the six most common PAEs by using a gas chromatograph-mass spectrometer (GC-MS). Of the 6 PAEs analyzed, only three PAEs, including di-n-butyl phthalate (DBP), di (2-Ethylhexyl) phthalate (DEHP), and diisononyl phthalate (DiNP) were detected in water samples. The total concentration of PAEs ranged from 1.44 to 12.08 µg/L, with a mean level of 4.76 µg/L. The total average concentration of PAEs found in the canal was higher than the criteria of 3 µg/L for PAEs recommended for the protection of fish and other aquatic organisms by the United States Environmental Protection Agency (USEPA). The results of the potential ecological risk assessment of the risk quotient (RQ) method revealed that DEHP and DiNP posed a high risk to algae and crustacean and crustacean and fish, respectively, whereas DBP posed a medium risk to the different aquatic species. However, current levels of noncarcinogenic and carcinogenic risks via ingestion and dermal exposure in children and adults are within acceptable limits. The baseline data of PAEs in this canal will be beneficial to the strategic and future pollutant control along the canal network.

## 1. Introduction

In the last few decades, the ubiquitous occurrence and ecotoxicological risk of phthalate acid esters (PAEs) in the aquatic environment have become a global issue. PAEs are mainly used as plasticizers in the production of plastics, rubber, polyvinyl chloride (PVC), and other polyethylene products to improve their flexibility, workability, and durability [1,2,3]. In addition, PAEs are used to improve the quality of a vast diversity of industrial, consumer, and personal care products [4]. PAEs residues have been frequently detected at measurable concentrations in aquatic ecosystems all over the world, due to high production and consumption volume, incessant release into the aquatic environment as well as their physicochemical properties [5,6]. Consequently, PAEs have been detected in the water phase ranging from 0.1 ng/L to 300 µg/L worldwide, indicating elevated PAEs pollution of the water phase in aquatic ecosystems [7,8]. PAEs contamination in the aquatic environments has been mainly attributed to the discharge of untreated and semi-treated wastewater from industrial and municipal activities, agricultural and aquaculture runoff, surface runoff from municipal solid waste sites, and atmospheric deposition into receiving water bodies [6,9]. Once in the aquatic environment, this group of organic chemicals can trigger adverse ecological effects on aquatic organisms at low concentrations, posing severe effects on the entire ecosystem [6].

In Thailand, elevated concentration of PAEs in the aquatic environment of 500 and 27.5 µg/L in drinking and surface water, respectively, have been reported [10,11]. Many studies have reported that continuous discharge of effluent from industrial and municipal wastewater plants into receiving water bodies are the main culprit of PAEs pollution of the aquatic environment [6,12,13,14,15]. However, to date, both industrial and municipal wastewater effluent is still being discharged into receiving water bodies in many developing countries, including Thailand [12,13,15,16,17]. Moreover, the legislation regulating the chemical pollution of the aquatic environment in Thailand does not specify PAEs [11].

The U-Tapao canal is a primary source of freshwater draining into the outer Songkla Lake, which is the largest natural lagoon in Thailand. The canal is the primary water resource for industrial usage, balancing the ecosystem, agriculture, aquaculture, and above all, for public water supply in Songkla Province, Southern Thailand. As a result of rapid economic development and urbanization in the region surrounding the water body, increasing industrial and municipal wastewater and effluents from agriculture and aquaculture have been discharged into the canal, which has resulted in changes in the water quality and ecology of the canal [16,17]. The canal frequently receives a large amount of industrial wastewater generated from industries including rubber, plastic, wood, and seafood at the rate of 41,000 m^3^ per day [18]. Besides, elevated concentrations of PAEs have been observed in industrial wastewater effluents that are frequently discharged into the U-Tapao canal [19].

Nevertheless, no study has evaluated the concentration of PAEs in surface water of the canal. Also, the ecological risk from PAEs in surface water of the canal to aquatic biota is mostly unknown, especially with regards to the risk posed to PAEs sensitive aquatic organisms of different taxonomic groups. Therefore, to protect the aquatic ecosystem of this primary freshwater source, it is imperative to determine the level of PAEs in water, as well as their subsequent ecological risk to aquatic biota.

Risk assessment involves assessing the risk to be encountered by sensitive organisms upon exposure to environmental concentrations of PAEs. The risk quotient (RQ) approach has been used to obtain deterministic ecological risk estimates of PAEs in water to sensitive aquatic biota, including algae, crustacean, and fish [13,15,20]. RQ for water is estimated by using the concentration of the individual PAEs and its critical toxicological endpoint for the test organism [21,22]. PAEs have been reported to pose significant threats to sensitive aquatic biota and the entire ecosystems [6,15]. A study demonstrated that di-n-butyl phthalate(DBP) and diethyl phthalate (DEP) have potential neurotoxicity to embryos of Zebrafish by inhibiting the activity of acetylcholinesterase [23]. Besides, PAEs have been reported to pose different levels of ecological risk to aquatic environments and that the different sensitivities of different aquatic species are neglected when assessing risk [24]. In risk assessment, assessing the potential health risks for humans derived from exposure to polluted environmental media is a significant approach applied for contaminants in water, sediment, soil, indoor and outdoor air, cosmetics, and food [25,26,27]. Assessing the noncarcinogenic and carcinogenic risk for humans depends on the routes of exposure to the PAEs [28,29,30]. Generally, the exposure pathways considered consist of dietary and non-dietary exposure routes [31]. Dietary exposures include the routine ingestion of food products and water contaminated with PAEs [28,30], while non-dietary exposures include dermal absorption via PAEs contaminated sediment and water; inhalation of residues stuck in the soil and accidental ingestion of contaminated soil [26,30,32]. Many studies have made known that prolonged exposure of PAEs may cause serious health problems including congenital disabilities such as reduced anogenital distance in baby boys, altered semen quality, hormonal and endocrine disruptions including early breast development in girls, shortened gestation, infertility, testicular dysgenesis, childhood social and mental impairment, obesity, asthma, and breast cancer [32,33,34,35,36]. Thus, there is a general concern that humans may be exposed to adverse effects of PAEs via the usage of polluted water and feeding on aquatic biota [35,36]. European Commission considered some PAEs congeners, including DBP, di-2-ethyl hexyl phthalate (DEHP), diisodecyl phthalate (DIDP), and di-isononyl phthalate (DINP) in the list of priority substances [5]. Besides, the United State Environmental Protection Agency (USEPA) has classified six PAEs as top priority pollutants, including four of the targeted PAEs in this study (DBP, benzyl butyl phthalate (BBP), DEHP, and di-n-octyl Phthalate (DnOP) [31]. Like other priority chemical substances, these synthetic industrial chemicals are subject to ecotoxicological risk assessment which are usually conducted in accordance to the Technical Guidance Documents [21,37,38]. Thus, it is imperative to monitor these PAEs in the environment, especially aquatic ecosystem [39]. Besides, the human health risk of the six targeted PAEs in this study from the U-Tapao canal is still unknown.

This study was carried out to (a) evaluate the PAEs levels in surface waters from the U-Tapao canal; (b) to assess the ecological risk of PAEs on sensitive aquatic biota by using the risk quotient method (RQ) to estimate the potential risk of detected PAEs congeners on three trophic levels of sensitive aquatic biota, including algae, crustacean, and fish; (c) to estimate the noncarcinogenic and carcinogenic risk of detected PAEs in contaminated water for children and adult via bathing and ingestion routes. The results from this study should be of great value in the risk control of PAEs in the U-Tapao canal, as well as provide scientific information on ecological and health risk of studied PAEs in aquatic ecosystems in developing tropical countries.

## 2. Methods and Materials

### 2.1. Study Site and Sampling

To assess the extent of phthalate acid ester contamination and potential risk in the aquatic ecosystem, a cross-sectional study was conducted in the U-Tapao canal in Songkhla Province, southern Thailand. The canal is one of the primary freshwater sources in southern Thailand. The U-Tapao canal is 68 km long and approximately 3 to 8 m deep with a flow rate that ranges < 6 in the dry season and ≥ 90 m^3^ in the wet period. The tropical monsoon climate of the canal is majorly influenced by the northeast and southwest monsoon with average rainfall ranging from 1600 to 2400 mm annually. The northeast monsoon causes heavy rainfall in the area from mid of October to mid-February. The temperature within and around the riverine ecosystem varies between 24 and 32 °C throughout the year. Land use pattern in the areas indicates that 75%, 15%, and 10% of the areas are covered by agricultural land, forest land, and urbanized areas, respectively.

**Figure 1:**
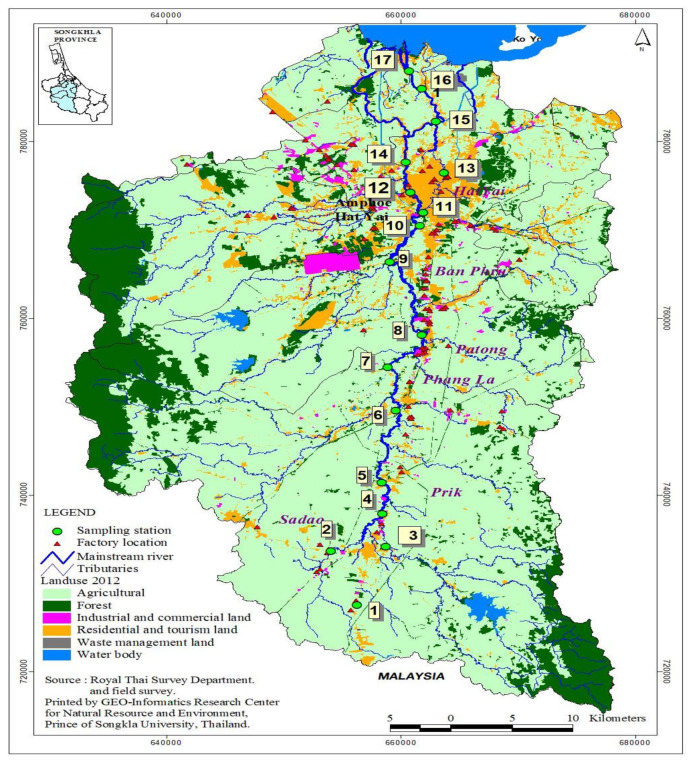
Map showing sampling sites for surface water of the U-Tapao canal. Source (Geoinformatics research center, Prince of Songkla University, 2019).

In this study, seventeen sampling sites for water were selected along the canal, from the upstream to downstream. The 17 sampling sites were classified into two different groups viz: urban and rural areas. Sampling sites in urban areas include ST1, ST2, ST3, ST4, ST6, ST7, ST9, ST10, ST12, and ST13. Sites located in the vicinity of the rural area were ST5, ST8, ST11, ST14, ST15, ST16, and ST17. Sampling sites in the canal are indicated in Figure 1. Water samples were collected from 17 sampling sites by using water sampler and transferred onto pre-treated brown bottles. The bottles were immediately placed on ice and were then kept at 4 °C in a laboratory refrigerator before analysis. All water samples were analyzed within three days.

### 2.2. Chemicals and Materials

Solvents used in this work include hexane, methanol, acetone, and dichloromethane, which were high-performance liquid chromatography (HPLC) grade, purchased from Waters, U.S.A. Phthalate standards included, di-n-butyl phthalate (DBP), benzyl butyl phthalate (BBP), di-2-ethyl hexyl phthalate (DEHP), di-n-octyl Phthalate (DnOP), di-isononyl phthalate (DiNP), and diisodecyl phthalate (DIDP) were purchased from AccuStandard, U.S. Solid-phase extraction cartridge Florisil (1 g 6 cc, Chrom and Sep), an internal standard of 100 mg/L of benzyl benzoate (BBZ) in n-hexane were all purchased from Dr. Ehrenstorfer Gmbh (Augsburg, Germany).

### 2.3. PAEs Pretreatment in Water

Water samples collected from the canal were pre-treated following already established protocol by [10,39] with slight modifications. Then, 1 L of each water sample was filtered through a 0.45 μm Millipore membrane. Solid-phase extraction (SPE) with an SPE cartridge, Florisil (1 g 6 cc, Chrom, and Sep) was carried out to extract the six targeted PAEs in water samples. The SPE cartridge was successively activated by 5 mL methanol/diethyl ether (5:95), volume/volume, 5 mL of methanol, and 10 mL of ultrapure water. The SPE cartridge was loaded with filtered water samples at a flow rate of 6–8 mL/min under vacuum. After extraction, the cartridge was dried under a vacuum for 5 min. Then, the cartridge was eluted by using 6 mL of methanol/diethyl ether; the eluent was placed into tubes. The eluate was evaporated to near dryness under a gentle nitrogen flow. The residue was redissolved with hexane to 1 mL, and benzyl benzoate as internal standard was added before the GC–MS analysis. The SPE cartridge (Florisil) used in this work was optimized and described in SI.

### 2.4. Instrumental Analysis by GC-MS

The analysis of DBP, BBP, DEHP, DnOP, DiNP, and DIDP contained in all the liquid extracts samples was performed by using a gas chromatograph/mass spectrometer (GC–MS), Agilent model 6890N GC–5973 MSD (Agilent Technologies), operating in electron impact (EI) and selective ion monitoring (SIM) modes with an HP-5 MS (30 m × 0.25 mm × 0.25 mm) fused-silica capillary column for chromatographic separation. The column temperature was initially set at 80 °C for 1 min, then raised to 280 °C at 15 °C and maintained for 1 min and increased up to 300 °C and held for 10 min. Helium gas (ultrahigh purity, 99.999%) was used as both carrier (1 mL/min) and makeup (50 mL/L) gas. Temperatures of the inlet (290 °C), quadrupole (150 °C), and ion source (250 °C) were used. Then, 1.0 µL liquid extracts were injected by automated samplers at 60 °C in splitless mode with a venting time of 0.75 min. The concentrations in the water were normalized to a dry weight (dw) basis.

### 2.5. Quality Assurance and Quality Control

To avoid background contamination of PAEs, sample contacts with plastic wares were avoided all through the entire procedure, and pre-treated laboratory utensils were cleaned with acetone three times before use. Five procedural blanks were extracted together with the water samples, all yielding non-detectable levels of the six targeted PAEs, signifying an absence of contamination all through sample treatment. Five different concentrations were used to prepare the calibration curve. The limit of detection (LOD) and limit of quantification (LOQ) for individual PAEs congeners were estimated based on a signal-to-noise ratio of 3 and 10 times, respectively. The percent recovery, precision (% RSD), LOD, and LOQ of the six targeted PAEs are shown in Table S3. The standard and sample chromatograms are shown in Figure S1. The validation of SPE and GC-MS methods used in this work were compared with previous studies and given in Table S4.

### 2.6. Ecological Risk Assessment

The risk quotient (*RQ*) model was used to evaluate the ecological risk of PAEs in this present study. As recommended by the European technical guidance document (TGD) on risk assessment of pollutants [21], *RQ* of individual PAEs congeners was calculated by dividing the measured environmental concentration (MEC) with the predicted no-effect concentration (PNEC) as indicated in Equation (1):

(1) $$RQ=\frac{\mathrm{MEC}}{\mathrm{PNEC}}.$$

In this work, the ecological risk of the PAEs measured in water was evaluated by calculating risk quotients (*RQs*) of individual PAEs congeners relative to algae, crustacean, and fish, as the representative of environmental living aquatic biota. The MEC value used was the mean concentration of individual PAEs detected in the water. The PNEC values used are indicated in Table S1. The ecological risk was grouped into three levels, including low risk, medium risk, and high risk. When the values of *RQ* > 1, high risk is expected, while values of 0.01 < *RQ* <1 indicate medium risks and values of *RQ* < 0.01 indicates a low risk [21,22]. The *RQ* method has already been applied in several studies dealing with the evaluation of the ecological risk of PAEs in water samples [20,22]. Table S1 shows the values used in the *RQ* calculation of the three PAEs congeners (DBP, DEHP, and DiNP) detected in the water.

### 2.7. Health Risk Assessment

In this present study, we conducted a human health risk assessment to ascertain whether the current levels PAEs congeners detected in the canal water samples may cause adverse health effects to humans. The approach used to evaluate the noncarcinogenic and carcinogenic risk of detected PAEs was described by USEPA [12,37,38]. The target population considered are adults and children, users of water from the canal. Assumptions of exposures pathways considered in this assessment include exposure through:

(i) Daily ingestion of drinking water from the canal.
(ii) Dermal contact through bathing with water from the canal.

Human exposure to toxic effects is expressed in terms of average daily dose (ADD), which is the number of pollutants taken into the human body daily during the exposure period calculated.

(2) $$ADD_{ingestion}=\frac{C\times \mathrm{IR}\times \mathrm{EF}\times \mathrm{ED}}{\left[\mathrm{BW}\right]\times \mathrm{AT}},$$

(3) $$ADD_{dermal}=\frac{DA_{event}\times \mathrm{SA}\times \mathrm{EV}\times \mathrm{EF}\times \mathrm{ED}}{\mathrm{BW}\times \mathrm{AT}}.$$

Non-cancerogenic effects

Hazard quotient (*HQ*) was conducted to assess the non-cancer effects of PAEs levels in humans. For oral or dermal exposures, the ADD was divided by reference dose (RfD), as shown in Equation (4). Any hazard quotient less than 1 is considered to be safe for lifetime exposure:

(4) $$HQ=\frac{\mathrm{ADD}}{\mathrm{RfD}}.$$

Hazard index (HI) of the different exposure scenarios, including oral ingestions and dermal pathway, were calculated by using Equation (5):

(5) $$\mathrm{HI}=\overset{n}{\underset{1\ldots n}{\sum }}HQ.$$

Carcinogenic risk

For DEHP, the only congener detected in water, which has the potential of causing cancer, the carcinogenic risk was estimated by using the Equations below:

(6) Risk=β×LADD,

(7) TR=∑R,

where:

*Risk* = potential cancer risk due to oral or dermal contact to PAEs contaminated water;
*LADD* = lifetime average daily dose exposure through oral or dermal exposure;
*β* = slope factor;
*TR* = total risk.

Regarding the noncarcinogenic risk indices, hazard quotient (HQ) or hazard index (HI) values > 1 indicates that the public health risk caused by the pollutant in the environmental matric is high, whereas values < 1 suggest acceptable risk. Cancer risk values surpassing 1 × 10^−4^ shows unacceptable health risks, while below 1 × 10^−6^ are considered to pose an acceptable health risk.

### 2.8. Data Analysis

Descriptive statistical parameters such as mean, standard deviation, maximum and minimum values were calculated to describe the concentrations of DEHP, DiNP, and DBP in the canal water by using SPSS version 2.0 (IBM). The graph was drawn by using Excel software 2016.

## 3. Results and Discussion

### 3.1. Environmental Concentrations of PAEs

Of the six targeted PAEs investigated in the U-Tapao canal, three congeners were detected, including DEHP, DBP, and DiNP. A statistical summary of PAEs levels in the surface water of the canal is shown in Table 1. Generally, outliers increase the variability in data, which decreases statistical power [40]. Consequently, excluding outliers can generate more statistically significant results [40]. In this present study, outliers were removed to obtain a more statistically reliable mean value. As indicated in Table 1, the total concentration of PAEs measured in surface water ranged from 1.44 to 12.08 µg/L, with DEHP constituting 44%, followed by DiNP (28.5%) and DBP (27.8%). The concentration of individual PAEs ranged from 1.28 to 5.28, non-detectable (ND) to 3.36, and ND to 3.44 µg/L for DEHP, DBP, and DiNP, respectively. DEHP was quantified in all the water samples, while DiNP and DBP were 65% and 47%, respectively. The mean concentration of individual PAEs, including DEHP, DiNP, and DBP were 2.51 ± 1.07, 2.12 ± 0.56, and 1.87 ± 0.85 µg/L, respectively. However, the mean environmental concentration of the total PAEs in the canal was 4.76 ± 2.81 µg/L, which was higher than the criteria of 3 µg/L of PAEs endorsed by the United States Environmental Protection Agency (USEPA) for the protection of fish and other aquatic biotas in the aquatic ecosystem [6,12]. Among individual PAEs, the mean value of DEHP was approximately 2-fold higher than the European Union environmental quality standards of 1.3 µg/L (DEHP), for the protection of the aquatic organism [6]. The order of PAEs abundance in the canal was found as DEHP > DiNP > DBP, which is consistent with [20].

**Figure 2:**
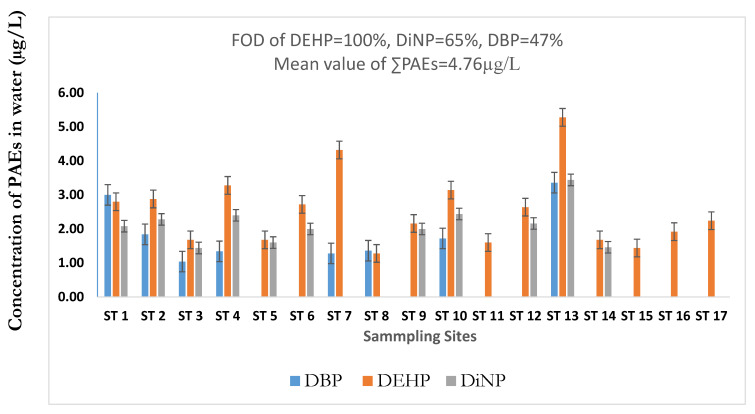
Distribution and Phthalate Esters (PAEs) concentrations in the Khlong U-Tapao canal.

As indicated in Figure 2, the distribution of PAEs in the canal shows that the total PAEs were recorded at the highest levels at sampling site (ST) 13 (12.08 µg/L dw), besides, among the individual PAEs, the highest concentration of DEHP, DiNP, and DBP were quantified up to 5.28, 3.44, 3.36 µg/L in site 13, respectively. This site (ST 13) is located at Hat Yai city, the most significant industrial and commercial city in southern Thailand. In addition, higher concentrations of PAEs were measured at ST 1, 2, 4, 7, and 10, located at Sadao, Prik, and Phang La cities. Most of these sampling sites are located near industrial and commercial areas. Thus, high levels of PAEs may be due to extensive industrial and commercial activities around these sampling sites. Industrial effluents and domestic wastewater generated in these areas are discharged into the canal at many points sources [16,17,18]. In case of low concentrations at sites 15, 16, and 17, since these sites are located in rural areas, industrial and domestic wastewaters are not expected to contribute significantly. However, effluents from agriculture and aquaculture activities and atmospheric depositions may be the potential sources of PAEs contamination in these locations. This is in agreement with previous observations [3,7]. Overall, PAEs levels found in urban areas were higher than the levels measured in rural areas, confirming the observation of previous studies that reported that urbanization influences the level of PAEs’ contamination of aquatic environments [6,8,15].

### 3.2. Comparison with Other Studies in Other Location

Although the complete data of PAEs congeners, including DBP, DEHP, BBP, and DnOP in water are routinely available, data for DiNP and DIDP are scarce. Nevertheless, the concentration of PAEs obtained in this study were compared with data of DBP, DEHP, and DiNP in water, documented in the published literature to evaluate the severity of the problem in the canal. As indicated by the data listed in Table 2, the total concentration of the 3 PAEs present in water was at a medium magnitude as compared to those measured in other locations [20,41,42,43,44,45]. The concentration of DEHP in the canal was comparable with those reported for the Bohai Sea and the Yellow Sea in China [43]. It was estimated to be 3-fold lower than those determined for Jiulong River and Jiulong River estuary [20,42,44], and approximately ranged from 2- to 20-fold higher than the concentrations documented for False Creek Harbor in Canada, coastal water of Sweden, and Songhua River basin, China [41,43,44]. Although the data of DiNP in water are limited for comparison, the concentration of DiNP in this study was compared with the few available studies. The concentration of DiNP was comparable with that of the Songhua River basin in China and was approximately 5–50 fold higher to those present in Bohai and the Yellow Sea, coastal water bodies in Sweden, Jiulong River, and Jiulong River estuary [40,43,44]. It is worthy of note that most of the available studies used for comparison of this grouping of PAEs congeners are current studies, except the case of False Creek Harbor in Canada, suggesting that detection of DiNP in the water phase was limited and may be due to scarcity of DiNP in previous studies. In contrast, DBP levels detected in this work were higher than those reported for other locations indicated in Table 2, except the Songhua River basin, China [20,41,42,43,44,45].

### 3.3. Ecological Risk of PAEs Congener on Sensitive Aquatic Species

Since aquatic ecosystems such as rivers, canals, and lakes receive industrial and municipal wastewater, urban and agricultural runoff, the continuous exposure of low doses of endocrine-disrupting compounds to resident aquatic biota cannot be evaded [46,47,48,49,50]. To estimate the potential adverse effects of the quantified PAEs in the investigated canal, surrogate species of three trophic levels of aquatic organisms, including algae, crustacean, and fish, were selected and treated separately in the estimation of the RQ for each measured PAEs. This was attributed to the scarcity of data on the aquatic toxicity of PAEs to sensitive native species.

The mean concentrations of individual PAEs in water were used to evaluate the potential ecological risk by RQ calculation. Table 3 depicts the RQ values of the DBP, DEHP, and DiNP in surface water of the U-Tapao canal. The RQ values of DBP were below 1 (0.01–1) for algae, crustacean, and fish, suggesting that the pollution status of DBP in the canal posed medium ecological risk on the aquatic environment based on these sensitive organisms. The RQ values of DEHP were above 1 for algae and crustacean and below 1 (0.01–1) for fish, which indicates that DEHP may pose a high risk on algae and crustacean, and medium risk on fish. RQ estimations for DiNP shows values were above 1 for crustacean and fish and below 1 (0.01–1) for algae, suggesting high risk on crustacean and fish, but the medium risk on algae. Overall, the result obtained in this study revealed that DEHP might pose the most significant adverse effects on algae species, while DiNP may pose the most significant risks on crustacean and fish. DBP may pose a medium risk across the three different species. Our findings were contrary to the negligible ecological risk of PAEs reported for the surface water of the Chao Phraya River in Thailand [48], because of the application of different evaluation methods. The risk ranking of the studied PAEs on aquatic biota in the canal was in the order of DEHP > DiNP > DBP, which is in agreement with the observation of [20].

PAEs have demonstrated endocrine-disrupting effects on different trophic levels of aquatic organisms, including algae, crustacean, and fish [6,49,50,51]. PAEs affect development in aquatic biota by altering both the thyroid hormone and growth hormone axes [51]. Besides, PAEs inhibit reproduction by interfering with cholesterol transport via the mitochondrial membrane, leading to a reduction of steroidogenesis. However, exposure to PAEs leads to the stimulation of peroxisome proliferator-activated receptors, the upsurge of fatty acid oxidation, and the reducing the ability to cope with the augmented oxidative stress, consequently, resulting in reproductive abnormalities including organ malformations, reproductive defects, and increased infertility [51]. The estrogenic effect of PAEs is the most critical adverse biological effect on sensitive aquatic biotas, including annelids mollusks, crustaceans, insects, fish, and amphibians, and found to interfere with the functioning of various hormone systems and induce genetic aberrations [50,51,52]. Several studies have reported the toxicity of PAEs to freshwater aquatic organisms at environmental concentrations [49,50,51,52,53]. PAEs, including DEHP, DBP, and other congeners, could damage the hemocytes of a freshwater prawn named *Macrobrachium* rosenbergii, at an environmental concentration of 100 µg/L [54]. Besides, DBP, DEHP, and DiNP posed adverse effects on the embryo of Zebrafish, with an LC_50_ of 0.63, 0.50 ppm, and leads to embryo mortality and typical toxicity symptoms, such as tail curvature, necrosis, cardiac edema, and no-touch response, in Zebrafish [53]. In addition, DEHP, DBP, and DiNP caused enhanced estrogenic activity at concentrations of 1.50 ppm in vitro and in vivo, suggesting that these PAEs congeners can induce the transactivation of ER in an additive manner [53]. A previous evaluation of the aquatic toxicity of PAEs on a freshwater crustacean showed that DEHP, DBP, and diethyl phthalate (DEP) had detrimental effects on fat metabolism, development, reproduction, and lifespan of Daphnia magna [55]. Though, sources have provided conflicting information on the potential aquatic toxicity of DiNP [5,56,57]. In the European Union (EU) risk assessment reports, DiNP was tentatively concluded not to cause adverse chemical effects towards the aquatic ecosystem [5]. However, it is essential to pay attention to the aquatic toxicity of DiNP because recent studies have reported that DiNP poses adverse effects on aquatic biota at environmental concentrations [55,56,57]. These necessitate the need to carry out further studies on the contamination status of DiNP in the aquatic ecosystem. Moreover, DBP has been found to induce acute and chronic toxicity in different freshwater organisms, including yellow perch *Perca* flavescens, the rainbow trout *Oncorhynchus* mykiss, the Nile tilapia *Oreochromis* niloticus, the mirror carp *Cyprinus* carpio, and the red killifish [58,59]. Overall, probable risks due to PAEs contamination may be expected in the aquatic wildlife of the U-Tapao canal.

### 3.4. Uncertainty Analysis of Ecological Risk

The adopting of RQ estimations, a deterministic method in conducting the ecological risk assessment in this study, will inevitably introduce uncertainty [12]. The usage of aquatic toxicity data obtained from non-native species introduced uncertainty due to the scarcity of toxicity data applicable for native species. Besides, endpoint toxicity data chosen in this study introduced uncertainty. This is because available acute toxicity data far outnumbered the reproductive toxicity data. However, the chronic and acute toxicity data used in assessing the ecological risk were derived from laboratory-based studies, contributing to uncertainty. This is because the laboratory observed aquatic toxicity values are assumed likewise to pose adverse effects on organisms living in a natural aquatic environment, thus, introducing a degree of uncertainty to all ecological risk assessments. RQ approach is a point estimate method; thus, it cannot provide detailed information on the qualitative or magnitude of ecological risks and cannot be used to establish a level of risk. Thus, RQ is useful as a screening tool that can help to focus on risk assessment. In addition, uncertainty came from a lack of data on the temporal and spatial variation of PAEs contamination status in the U-Tapao canal. Further study should be conducted to get more PAEs exposure data in a wide range of temporal and spatial scales. Thus, a more accurate ecological risk assessment of risks will be conducted. Besides, this study did not consider PAEs levels in suspended particles and sediment. Other pollutants, including pesticides, pharmaceuticals, and other endocrine-disrupting chemicals, were also not considered. Given sufficient concentrations, those contaminants are likely to influence the cumulative toxicity to the aquatic biota significantly. All the above factors suggest that the numerical results obtained from the ecological risk assessment in this and similar studies should be interpreted with caution.

### 3.5. Human Health Risk

Aquatic environmental media, including water and organisms, are susceptible to the presence of PAEs beyond a certain level, and their consumption can affect human health [6,13,15]. Therefore, it is imperative to tell the health status of the U-Tapao canal water. The probable carcinogenic and noncarcinogenic health risk through ingestion and dermal exposure pathways for both children and the adults were calculated by using the exposure factors in Table S2. Exposure and potential risk values are presented in Table 4, Table 5 and Table 6. According to Table 4, the values of the noncarcinogenic effects of PAEs in adults were 3.74 × 10^−2^ (DBP), 2.51 × 10^−1^ (DEHP), and 3.69 × 10^−2^ (DiNP), via ingestion while dermal absorption values were 2.06 × 10^−3^ (DBP), 3.44 × 10^−2^ (DEHP), and 5.84 × 10^−3^ (DiNP). In addition, the total noncarcinogenic risk in adults via oral and dermal were 3.25 × 10^−1^ and 9.49 × 10^−2^, respectively. Table 5 shows that in children, the noncarcinogenic health effects values via ingestion routes were 1.87 × 10^−2^ (DBP), 1.26 × 10^−1^, (DEHP), and 1.84 × 10^−2^, (DiNP), however, the values via dermal contact were 2.14 × 10^−3^ (DBP), 3.59 × 10^−2^ (DEHP), and 6.09 × 10^−3^ (DiNP). Besides, the total noncarcinogenic (HI) risk of DBP, DEHP, and DiNP via oral and dermal exposure pathways in children were 1.63 × 10^−1^ and 4.13 × 10^−2^, respectively. The noncarcinogenic results for both children and adults were below 1, indicating that PAEs cannot cause adverse health effects on adults and children. As indicated in Table 6, the probable cancer risk values of DEHP were 3.01 × 10^−5^, via ingestion, and 4.13 × 10^−6^ through dermal adsorption in adults as a result of using water from the canal, whereas in children, the cancer risk values of DEHP were 3.01 × 10^−6^ via ingestion pathway and 8.59 × 10^−7^ through dermal contact. In addition, the total cancer risk of DEHP values via the two-exposure pathway considered in this study for adult and children were 3.42 × 10^−5^ and 3.87 × 10^−6^, respectively. These results suggested that adults and children may not be subjected to cancer risks as a result of oral or dermal exposure to contaminated water. Generally, the noncarcinogenic risk (HQ) or (HI) values >1 indicate that the assessed pollutant posed adverse effects on public health and suggests further monitoring and assessment study. Based on the result obtained in this present study, exposure of humans to PAEs in water samples from the canal, via oral and dermal routes was acceptable and did not pose a public health risk. Our result is in agreement with previous studies [13,29], where PAEs were not observed to pose a health risk to humans via exposure through surface water. Nevertheless, many studies have reported that some PAEs are environmentally active endocrine-disrupting pollutants, which can cause toxic and cancer effects at very low concentrations [60,61,62,63,64,65]. Although exposure to PAEs in our study was safe in terms of toxic (noncarcinogenic) and carcinogenic effects, assessment of contaminants from other sources, including indoor and outdoor air, cosmetics, drinking water, and food, is essential for determining the actual cumulative exposure [13,60]. Besides, the risk assessment approach used in this present study is only a deterministic approach, which presented a quantitative estimation of the human health risk. Therefore, future research should aim to monitor endocrine-disrupting pollutants in drinking water, outdoor and indoor air, cosmetics, and food. Additionally, a probabilistic risk assessment method could be used to obtain a qualitative insight into the potential health risk.

Although the carcinogenic risk of DEHP obtained in this study was considered safe for both adults and children, nevertheless, environmental exposures of humans to DEHP and its primary metabolites have been associated with cancer risk. Studies have proposed that the carcinogenic effects of DEHP in humans and animals are mediated via multiple molecular signals, including DNA damage [61,65]. The carcinogenic effects of DEHP are found in different target tissues, notably in the liver and testis [62]. The primary metabolite of DEHP, mono-2-ethyl hexyl phthalate (MEOH), has been observed to accelerate the progression of prostate cancer by activating the Hedgehog signaling pathway [63]. Besides, there are emerging reports describing the toxicity of PAEs at levels relatively lower than reference doses (RfD) by using in vitro models. Low concentrations ranging from 10^−5^ to 10^−8^ mol/L, DEHP, BBP, and DBP induced proliferation in human breast cancer via the PI3K/AKT signaling pathway as well as displayed estrogenic effects [64]. Moreover, an epidemiological survey reported that monoethyl phthalate (170 µg/g creatinine) was positively associated with the prevalence of breast cancer in women patients of northern Mexico [66]. The surface water samples with the highest and lowest health risks were found in sites ST13 and S17, respectively.

### 3.6. Uncertainty Analysis of Health Risk

All risk estimations methods involve some degree of uncertainty that exists at several levels. The two primary sources of uncertainty in the assessment of human exposures to pollutants include the concentrations of the assessed pollutants to which the potential population may be exposed all through the exposure period and ambiguity about the exposed population [12]. In this present study, both of these influenced the result significantly. The real exposed population could not be studied; thus, the health risk was based exclusively on hypothetical exposure scenarios. Consequently, uncertainty in the results obtained may be significant. Besides, uncertainty in dose-response of both noncarcinogenic and carcinogenic data was also a contributory factor. The reference dose values employed herein have uncertainty factors of 1 or 2 orders of magnitude. The various assumptions used in this study, such as the lifetime of 70 years, bodyweight of 60 kg for adults, and 20 kg for children, increase the uncertainties involved in the assessment process. Thus, the results provided in this work are simply an indication of the potential health risk associated with potential exposure to canal surface waters if used for drinking and bathing purposes. It is likely that these risks predicted may be an underestimation of the actual health risks as they only portray the risks associated with a population exposed to the detected 3PAEs congeners in this work. No other pollutants in the canal were considered in this present study; nevertheless, the likelihood of the occurrence of other pollutants that are injurious to humans in the canal water is high. Therefore, a health risk may be higher than what is represented in this paper.

## 4. Conclusions

A cross-sectional survey of PAEs in surface water of the U-Tapao canal was conducted, and the ecotoxicological risks of quantified PAEs were then evaluated. The mean environmental concentration of individual congeners was 1.87, 2.12, and 2.51 µg/L for DBP, DiNP, and DEHP, respectively. The mean concentration of DEHP measured in the water sample was approximately 2-fold higher than environmental quality standards (EQS) of DEHP in surface water, recommended for the protection of the aquatic ecosystem. The spatial distribution of PAEs in the investigated canal revealed that urbanization influences PAEs’ distribution along the canal network. Preliminary results of the ecological risk assessment using the RQ method revealed that DEHP and DiNP exhibited high risk, while DBP showed the medium risk to the aquatic system in the investigated canal. In addition, DEHP poses the most significant adverse effects on algae species, while DiNP poses the most significant risks on crustacean and fish, and DBP poses medium risk across the three different species. However, the deterministic approach of noncarcinogenic risk and carcinogenic risk caused by PAEs detected in water samples of the canal, through oral and dermal exposure in children and adults, revealed that the current levels of noncarcinogenic risks and carcinogenic risks through these two means of exposure are within acceptable limits.

## Figures and Tables

**Table 1:** The concentration of Phthalate esters (PAEs) in surface water of U-Tapao canal (µg/L).

SITES	Latitude	longitude	DBP	DEHP	DiNP	∑PAEs
ST 1	7.108381	100.465011	3.00	2.80	2.08	7.88
ST 2	7.002145	100.455991	1.84	2.88	2.28	7.00
ST 3	6.979739	100.463408	1.04	1.68	1.44	4.16
ST 4	6.596520	100.486966	1.34	3.28	2.40	7.02
ST 5	6.639564	100.436129	ND	1.68	1.60	3.28
ST 6	6.602124	100.406920	ND	2.72	2.00	4.72
ST 7	6.635161	100.393411	1.28	4.32	ND	5.60
ST 8	6.673192	100.433361	1.36	1.28	ND	2.64
ST 9	6.705086	100.433163	ND	2.16	2.00	4.16
ST 10	6.779266	100.443868	1.72	3.14	2.44	7.30
ST 11	6.823206	100.437958	ND	1.60	ND	1.60
ST 12	6.856377	100.464485	ND	2.64	2.16	4.80
ST 13	6.931202	100.439884	3.36	5.28	3.44	12.08
ST 14	6.979740	100.463409	ND	1.68	1.46	3.14
ST 15	7.033356	100.452362	ND	1.44	ND	1.44
ST 16	7.075167	100.475782	ND	1.92	ND	1.92
ST 17	7.126859	100.455496	ND	2.24	ND	2.24
Mean			1.87	2.51	2.12	4.76
SD			0.85	1.07	0.56	2.81
Minimum			ND	1.28	ND	1.44
Maximum			3.36	5.28	3.44	12.08
Detection Frequency			47	100	65	100

**Table 2:** Comparison of the concentrations of di-n-butyl phthalate (DBP), di-2-ethyl hexyl phthalate (DEHP), di-isononyl phthalate (DiNP) DEHP, DBP, and DiNP in surface water of other location.

S/N	Location	DBP (µg/L)	DEHP (µg/L)	DiNP(µg/L)	References
1	Bohai and Yellow Sea, China	0.27–1.24	0.062–4.35	ND–0.054	[43]
2	Jiulong River estuary China	0.30–1.77	0.12–12.40	ND–0.52	[44]
3	Jiulong River, China	0.28–2.40	0.79–10.90	ND–524	[20]
4	Songhua River basin, China	0.19–4.76	0.36–2.68	ND–2.47	[42]
5	Coastal waters, Sweden	<0.19–0.49	<0.068–0.22	<0.05–0.13	[41]
6	False Creek Harbor, Canada	0.050–0.24	0.170–0.44	0.061–0.14	[45]
7	U-Tapao canal, Thailand	ND–3.36	1.28–5.28	ND–3.44	This study

**Table 3:** Risk quotient (RQ) values of 3 PAEs to sensitive aquatic species.

PAEs	Algae	Crustacean	Fish
DBP	0.09	0.07	0.19
DEHP	25.10	2.98	0.42
DiNP	0.12	6.24	5.05

**Table 4:** *HQ* values of exposure to PAEs via ingestion and dermal contacts in adults.

PAEs	Mean (µg/L)	ADDing (mg/kg/day)	RfD (mg/kg/day)	HQ	ADDdm (mg/kg/day)	RfD (mg/kg/day)	HQ
DBP	1.87 ± 0.85	3.74 × 10^−3^	1.0 × 10^−1^	3.74 × 10^−2^	2.06 × 10^−4^	1.0 × 10^−1^	2.06 × 10^−3^
DEHP	2.51 ± 1.07	5.02 × 10^−3^	2.00 × 10^−2^	2.51 × 10^−1^	6.88 × 10^−4^	2.00 × 10^−2^	3.44 × 10^−2^
DiNP	2.12 ± 0.56	4.24 × 10^−3^	1.15 × 10^−1^	3.69 × 10^−2^	6.72 × 10^−4^	1.15 × 10^−1^	5.84 × 10^−3^
HI				3.25 × 10^−1^			9.49 × 10^−2^

**Table 5:** *HQ* values of exposure to PAEs via ingestion and dermal contact in children.

PAEs	Mean (µg/L)	ADDing (mg/kg/day)	RfD (mg/kg/day)	HQ	ADDdma (mg/kg/day)	RfD (mg/kg/day)	HQ
DEHP	2.51 ± 1.07	2.51 × 10^−3^	2.00 × 10^−2^	1.26 × 10^−1^	7.17 × 10^−4^	2.00 × 10^−2^	3.59 × 10^−2^
DiNP	2.12 ± 0.56	2.21 × 10^−3^	1.15 × 10^−1^	1.84 × 10^−2^	7.00 × 10^−4^	1.15 × 10^−1^	6.09 × 10^−3^
HI				1.63 × 10^−1^			4.13 × 10^−2^

**Table 6:** Risk values of carcinogenic risk.

Age Group	PAE	LADD Ingestion	Slope Factor	Cancer Risk	LADD Dermal	Slope Factor	Cancer Risk	TR
Adult	DEHP	2.15 × 10^−3^	1.40 × 10^−2^	3.01 × 10^−5^	2.95 × 10^−4^	1.40 × 10^−2^	4.13 × 10^−6^	3.42 × 10^−5^
Children	DEHP	2.15 × 10^−4^	1.40 × 10^−2^	3.01 × 10^−6^	6.14 × 10^−5^	1.40 × 10^−2^	8.59 × 10^−7^	3.87 × 10^−6^

## Supplementary Materials

The following are available online at https://www.mdpi.com/2305-6304/8/3/58/s1, Figure S1: Chromatograph of DBP, BBP, DEHP, DnOP, DiNP and DIDP, Table S1: Toxicity of PAEs in some sensitive aquatic organisms, Table S2: Exposure factors used for health risk assessment, Table S3: QA/QC parameters for the extraction and analysis of six targeted PAEs, Table S4: comparing the validation of SPE and GC-MS methods used in this study with previous studies.
