# Supplementary material for: Occurrence, Ecological and Health Risk Assessment of Phthalate Esters in Surface Water of U-Tapao Canal, Southern, Thailand
Source: Toxics. 2020 Aug 17;8(3):58. doi: 10.3390/toxics8030058 (PMC7560390; doi:10.3390/toxics8030058)
Supplement: Supplementary file 1 [file toxics-08-00058-s001.pdf]

toxics

Supplementary Material: Occurrence, Ecological and Health Risk Assessment of Phthalate Esters in Surface Water of U-Tapao Canal, Southern, Thailand

Okpara Kingsley [1,2,*] and Banchong Witthayawirasak [1,2]

1. Ecological risk assessment

The PNEC values used were obtained from median lethal concentration (LC_{50}) or median effects concentration (EC_{50}) divided by an assessment factor (A.F.) of 1000 when short-term/acute toxicity data were available. Alternatively, PNEC values were also obtained by using long-term/chronic no effect concentration (NOEC) values for 1, 2, and 3 trophic levels divided by an A.F. of 100, 50, and 10, respectively (European Commission, 2003). Short-term or long-term toxicity data of PAEs to aquatic biota, including algae, crustaceans, and fish, were collected from the USEPA ECOTOX database (http://cfpub.epa.gov/ecotox) and published articles. The PNEC values obtained based on these toxicity data are shown in Table S1. The ecological risk was group into 3 levels, including low risk, medium risk, and high risk. When the values of R.Q.>1, high risk is expected, while values of 0.01< R.Q. <1 indicate medium risks and values of R.Q. <0.01 indicates a low risk [1, 2, 3]. R.Q. method used in this study has been applied in previous studies dealing with the evaluation of the ecological risk of PAEs in water samples [3–4]. Table 1 shows the values used in the R.Q. calculation of the three PAEs congeners (DnBP, DEHP, and DiNP) detected in the water.

Table S1 Toxicity of PAEs in some sensitive aquatic organisms.

PAEs Congeners	Population	Species	Toxicity data (μg/L)	AF	PNEC (μg/L)
DnBP	Algae	Pseudokrichneriella subcapitata	96 h, population, NOEC = 210	10	21
	Crustaceans	Americamysis bahia	21 d, mortality, NOEC = 260	10	26
	Fish	Oncorhynchus mykiss	99 d, growth, NOEC =100	10	10
DEHP	Algae	Pseudokirchneriella subcapitata	96 h, population, EC50 = 100	1000	0.1
	Crustaceans	Mytilus edulis	21 d, mortality, NOEC = 42	50	0.84
	Fish	Gasterosteus aculeatus	28 d, mortality, NOEC = 300	50	6
DiNP	Algae	Selenastrum capricornutum	120 h, population NOEC = 1800	100	18[a]
	Crustacean	Daphnia magna	21 d, mortality, NOEC = 34	100	0.34
	Fish	Danio rerio	21day, reproduction NOEC = 42	100	0.42[b]

NOEC: no observed effect concentration; A.F.: assessment factor; EC50: median effect concentration. [a]: [9]; [b]: [5].

Table S2 Exposure factors used for health risk assessment.

Symbol	meaning	units	Value	Reference
E.F.	Exposure frequency	Days/year	365	[13]
ET	Exposure time	Min/event	12[a] 6[c]	[8]
EVF	Event frequency	Event/day	1	[13]
E.D.	Exposure duration	year	NCR = 30years<br>CR = 70 years<br>6[c]	[13]
I.R.	Ingestion rate	Liter/day	1L[c] 2L[a]	[6]
BW	Body weight	kg	20[c] 60[a]	[7]
AT	Averaging time		NCR= 10950<br>CR = 25550	[13]
S.A.	Skin surface area	Cm^2	5700[a] 2800[c]	[8]
DAF	Dermal absorption factor	unitless	0.1	[8]
RfD	Reference dose		DEHP = 0.02<br>DnBP = 0.1<br>DiNP = 0.115	[13]
SF	Cancer slope factor	Mg/kg/d	DEHP = 0.014	[13]

Table S3 QA/QC parameters for the extraction and analysis of six targeted PAEs.

PAEs	Linearity R^2	Target ion	Retention time (min)	Recovery (%) *n* = 3	RSD (%)	LOQ *n* = 7 µg/L	LOD *n* = 7 µg/L
DBP	0.999	223, 205, 167	7.57	84	5.9	0.88	0.32
BBP	0.999	205, 149, 91	8.77	69	6.2	0.78	0.12
DEHP	0.999	279,167, 149	9.29	99	7.2	0.98	0.45
DnOP	0.999	279,261, 149	9.84	93	6.8	0.89	0.42
DiNP	0.999	293,127	9.93	110	7.6	1.02	0.84
DIDP	0.999	307,141	10.44	97	8.4	1.82	1.04

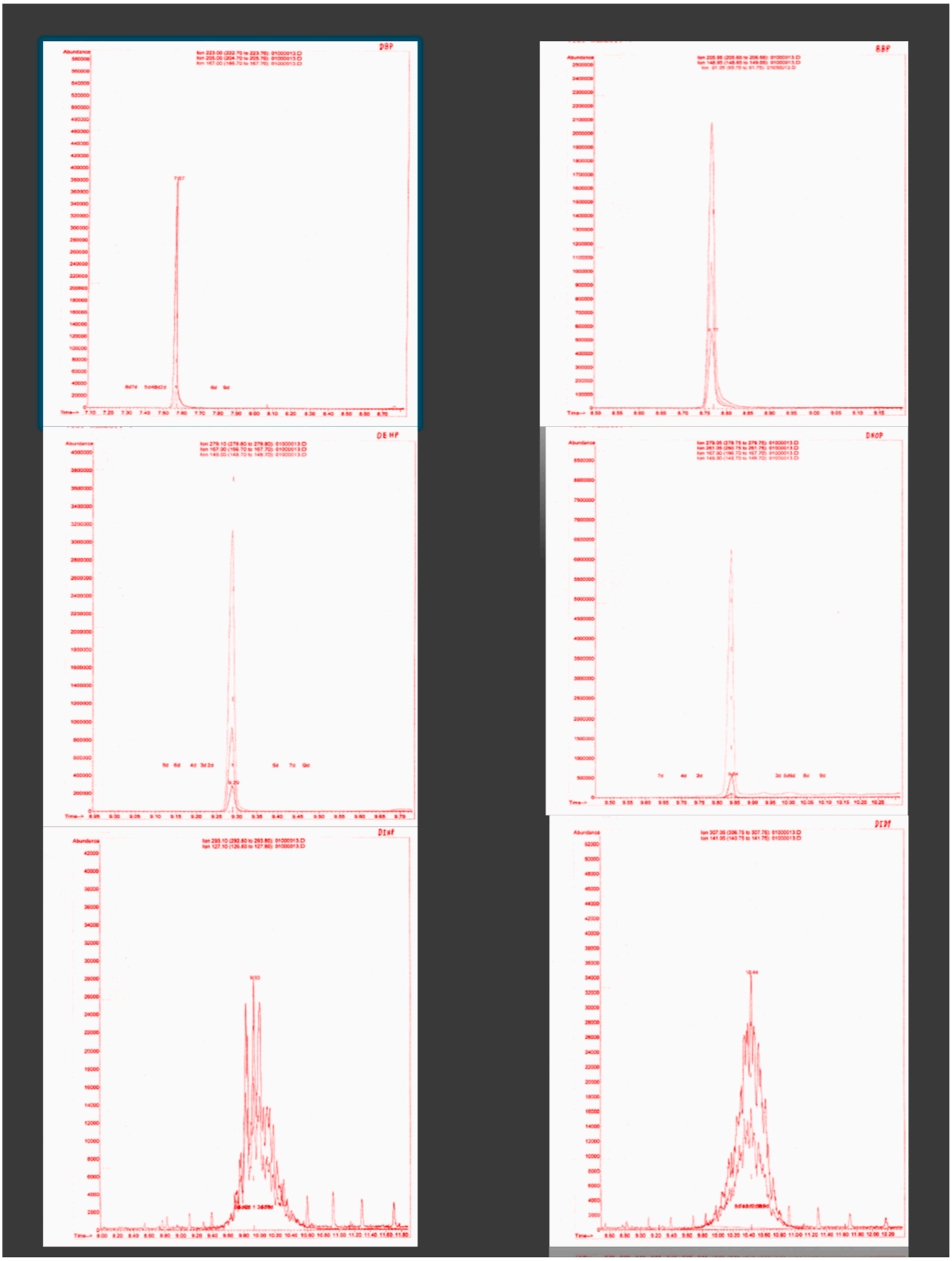

Figure S1. of DBP, BBP, DEHP, DnOP, DiNP and DIDP.

Table S4. comparing the validation of SPE and GC-MS methods used in this study with previous studies.

Analytes	Pretreatment (Solid phase extraction)	Analytical system	Analytical column	Analytical characteristic	Reference
DBP, DEHP, DEP, DMP	SPE, Florisil	GC-MS	DB-5 (30 m, 0.25 mm id, 0.25µm)	LOD: 0.25–0.50µg/L<br>LOQ: 0.08–0.17 µg/L<br>Recovery: 95–110%<br>RSD: 1.20–2.60%	[17]
11 PAEs including DnBP, BBP, DEHP, DnOP, DiNP and DIDP	SPE (Fe3O4@pDA NPs)	GC-MS	HP–5 ms; (15 m × 0.25 mm, 0.25µm)	LOD:0.36–4.20 µg/L<br>LOQ:9–20<br>Recovery: 71–117 %<br>RSD:3–18%	[16]
6 PAEs including DEHP, BBP, DBP, DEP DMP,DnOP	SPE, Florisil	GC-ECD	DB-5(30m × 0.53mmid, 5%phenyl/95%methyl silicone)	LOD:0.049–0.640 µg/L<br>LOQ:NR<br>Recovery:89.4–102%<br>RSD:3.8–17.7%	[10]
DEHP, BBP, DBP, DEP DMP,DnOP	SPE (Florisil	GC-MS	*Hp* -5(30 m × 0.25 mm x 0.25 pm)	LOD:0.05–0.10 µg/L<br>LOQ:<br>Recovery:72–95%<br>RSD:	[12]
DBP, BBzP, DEHP, DnOP, DiNP, DiDP	SPE	GC–ECD	DB-5(30m × 0.53mmid, 5%phenyl/95%methyl silicone)	LOD:0.39–0.94 µg/L<br>LOQ:<br>Recovery: 89–113%<br>RSD:6.87–17.34%	[14]
DEP, DIBP, DBP, DEHP, BBP, DNOP	SPE	GC-MS	HP-5MS (30 m × 0.25 mm I.D.; 0.25 µm film).	LOD: 0.010–0.056 µg/L<br>LOQ:0.035–0.19 µg/L<br>Recovery: 88–110%<br>RSD: < 8.5%	[15]
DMP, DEP, DBP BBP, DEHP,DnOP	SPE (OASIS HLB)	GC-MS	(30 mm × 0.32 mm × 0.11 mm, Varian)	LOD:0.002–0.010 µg/L<br>LOQ:NA<br>Recovery:55–95%<br>RSD:<15%	[16]
DnBP, BBP, DEHP, DnOP, DiNP and DIDP	SPE(Florisil)	GC-MS	HP-5 MS (30 m × 0.25 mm × 0.25 mm)	LOD: 0.12–1.04 µg/L<br>LOQ:0.78–1.82 µg/L<br>Recovery:69–110%<br>RSD:5.9–8.4%	This present study

2. Optimization of the SPE Cartridges

Response: Prior to extraction, the SPE columns were conditioned with 5.0 mL methanol under vacuum, then followed by 5.0 ML with ultra-pure water. 100 mL of ultra-pure water was spiked with standard solution of PAEs (20 mg/L, each), and subsequently passed through the column with the flow rate of 1.0 mL. PAEs were eluted with various organic solvent. The percentage recovery was determined by using GC. The limit of detection of the SPE were taken as the lowest concentration of PAEs that could be extracted and yielding good recoveries. The recoveries of the six PAEs in the spiked samples ranged 69 to 110% with relative standard deviation (RSD) values ranging from 5.9 to 8.4% (Table S3).

References

Toxics **2020**, *8*, 58 4 of 5

1. European commission, "Technical Guidance Document on risk assessment in support of Commission Directive 93/67/EEC, Commission Directive 98/8/EC, Commission Regulation (EC) No 1488/94, Commission Directive 93/67/EEC. ," 2003.
2. Li, J.; Liang, Z.; Gong, N.; Zhang, R.; Duan, H. Occurrence, spatial distribution, historical trend and ecological risk of phthalate esters in the Jiulong River, Southeast China. *Sci. Total Environ.* **2017**, *580*: 388–397.
3. Chen, H.; Mao, W.; Shen, Y,; Feng, W.; Mao, G.; Zhao, T.; Yang, L.; Yang, L.; Meng, C.; Li, Y.; Wu, X. Distribution , source , and environmental risk assessment of phthalate esters (PAEs) in water , suspended particulate matter , and sediment of a typical Yangtze River Delta City , China. Environ Sci and Pollut Res. **2019**, *26*, 24609–24619.
4. Ramzi, A.; Gireeshkumar, T.R.; Rahman, K.H.; Balachandran, K.K.; Shameem, K.; Chacko, J.; et al. Phthalic acid esters – A grave ecological hazard in Cochin estuary, India. Marine Pollution Bulletin. **2020**,*152*.
5. Santangeli, S.; Maradonna, F.; Zanardini, M.; Notarstefano, V.; Gioacchini, G.; Forner, P.I.; Habibib, H.; Carnevali, O. Effects of diisononyl phthalate on Danio rerio reproduction. *Environ. Pollut.* **2017**, *231*, 1051–62.
6. Hensawang, S.; Chanpiwat, P. Health impact assessment of arsenic and cadmium intake via rice consumption in Bangkok, Thailand. *Environ. Monit. Assess* **2017**, *189*: 599.
7. Fatoki, O.S.; Bornman, M.; Ravandhalala, L.; Chimuka, L.; Genthe, B.; Adeniyi, A. Phthalate ester plasticizers in freshwater systems of Venda, South Africa and potential health effects. *Environ. Pollut*. **2017**, *231*, 1051–62.
8. Olujimi, O.O.; Aroyeun, O.A.; Akinhanmi, T.F.; Arowolo, T.A. Occurrence, removal and health risk assessment of phthalate esters in the process streams of two different wastewater treatment plants in Lagos and Ogun States, Nigeria. *Environ. Monit. Assess*. **2017,** *189*, 345.
9. Staples CA, Adams WJ, Parkerton TF, Gorsuch JW, Biddinger GR, Reinert KH. Environmental Toxicology Review Aquatic toxicity of eighteen phthalate esters. Environ Toxicol Chem. 1997.
10. Ebitson, M. and Holmes, B. Determination of Trace Phthalate Ester Compounds for EPA Method 8061A no. 603, 2011.
11. González, S.J.; Socas, R.B.; Hernández, B.J.; and Rodríguez, D.M.; Determination of phthalic acid esters in water samples using core-shell poly(dopamine) magnetic nanoparticles and gas chromatography tandem mass spectrometry *J. Chromatogr. A*, **2017**.
12. Holadová, K.; Hajšlová, J. A Comparison of Different Ways of Sample Preparation for the determination of Phthalic Acid Esters in Water and Plant Matrices. *International Journal* **2013**, 37–41.
13. USEPA (United States Environmental Protection Agency) MidAtlantic Risk Assessment. Regional Screening Level (RSL) Summary Table. Washington DC. MAY 2013.
14. Zhang, A.J.; Perati, P.; and Lopez,L. Automated Solid-Phase Extraction of Phthalates for Drinking Water Samples.
15. Ye, Q.; Liu, L.; and Chen, Z.; Analysis of phthalate acid esters in environmental water by magnetic graphene solid phase extraction coupled with gas chromatography – mass spectrometry. *J. Chromatogr. A* **2014**, *1329*, 24–29.
16. Liu, X,, Shi, J.; Bo, T.; Zhang, H.; Wu, W.; and Chen, Q. Occurrence of phthalic acid esters in source water: A nationwide survey in China during the period of 2009 e 2012, *Environ. Pollut.,* **2014**, *184*, 262–270.
17. Kanchanamayoon, P.P.W. Determination of Phthalate Esters in Drinking Water Using Solid-Phase Extraction and Gas Chromatography. *J. Appl. Sci.,* **2010**, *17*, 1987–1990.
